# The effect of preoperative anxiety level on mean platelet volume and propofol consumption

**DOI:** 10.1186/s12871-020-0955-8

**Published:** 2020-02-01

**Authors:** Ali İhsan Uysal, Başak Altıparmak, Melike Korkmaz Toker, Gülseda Dede, Çiğdem Sezgin, Semra Gümüş Demirbilek

**Affiliations:** 1 Department of Anesthesiology and Reanimation, Muğla Sıtkı Koçman University Training and Research Hospital, Muğla, Turkey; 2 Department of Anesthesiology and Reanimation, Muğla Sıtkı Koçman University, Muğla, Turkey

**Keywords:** Anxiety, Mean platelet volume, Propofol consumption

## Abstract

**Background:**

The mean platelet volume (MPV) is an important indicator of platelet function with large platelets showing higher enzymatic and metabolic activity than other platelets. There can be a relationship between increased platelet activity and anxiety and depression. Our primary hypothesis was that patients with high anxiety scores would have higher MPV, and the secondary hypothesis was that propofol induction time and total propofol consumption within the first 30 min of surgery would be higher in patients with higher anxiety scores.

**Methods:**

The Beck Anxiety Inventory (BAI) was administered to the participating patients 1 day before surgery to evaluate the level of anxiety. Based on the scores from the BAI, 40 patients with an anxiety score of < 8 were assigned to the non-anxious group (Group NA) and 40 patients with an anxiety score of ≥8 were assigned to the anxious group (Group A). At the anesthesia induction the mean time to achieve an entropy value below 60 (T1) was recorded. The total intraoperative propofol consumption within the first 30 min was recorded.

**Results:**

There was a statistically significant difference between the groups in terms of preoperative MPV and demographic data, including age and sex. The mean total propofol consumption at 30 min after induction in the groups was statistically significant. The cut-off value for MPV was calculated as 9.65.

**Conclusions:**

The preoperative MPV values and propofol consumption at 30 min among patients with high preoperative anxiety scores were high. We suggest that MPV is helpful in the clinical practice in predicting the amount of anesthetic agents required for the 30 mins of anesthesia.

## Background

Anxiety is an unpleasant feeling of apprehension, irritability, and strain. An individual experiencing anxiety gives a dramatic neuroendocrine response which increases cardiovascular activity and metabolism [1]. Such neuroendocrine responses result in an increase in catecholamine levels, sympathetic activity, and cortisol secretion [2].

Serotonin is an important factor in the psychopathology of anxiety disorders. Impaired serotonin (5-HT) function due to the hyperserotonergic state may lead to anxiety and fear response stimulated by amygdala [3]. Serotonin also plays a key role in the regulation of vascular tone on the vessel wall and platelet aggregation [4]. Peripheral platelet models reflect central serotonergic function and are commonly used as an indicator of a central 5-HT metabolism. Accordingly, platelets can be considered a marker of biochemical alterations occurring in the brain in the presence of anxiety [5]. The mean platelet volume (MPV) is an important indicator of platelet function with large platelets showing higher enzymatic and metabolic activity than other platelets [6].

Previous studies have evaluated the effects of anxiety on the time required for the induction of anesthesia with propofol and propofol consumption [7, 8]; however, there has been no study, to date, evaluating the effects of MPV on propofol induction time and propofol consumption. In the present study, we aimed to evaluate the possible relationship between preoperative anxiety, as assessed by the Beck Anxiety Inventory (BAI), 1 day prior to surgery and MPV. Our primary hypothesis was that patients with high anxiety scores would have higher MPV, and the secondary hypothesis was that propofol induction time and total propofol consumption within the first 30 min of surgery would be higher in patients with higher anxiety scores.

## Methods

After obtaining the approval of the Clinical Trials Ethics Committee of Muğla Sıtkı Koçman University and written informed consents from each patient, a screening was performed to evaluate anxiety levels in the participating patients. The study included patients from both sexes within an age range of 18 to 65 years, and at American Society of Anesthesiology (ASA) physical status I–II who were scheduled for elective laparoscopic cholecystectomy. Patients on psychotropic medication due to a psychiatric disease, patients with a neurological disorder, pregnant and breastfeeding women, patients with uncontrolled hypertension, metabolic disease or hematological disorders, patients with hypercholesterolemia and those who received medication for a chronic condition within the past 2 months, and patients with a chronic cardiac, respiratory or renal disease were excluded. The study was conducted in accordance with the principles of the Declaration of Helsinki.

The Beck Anxiety Inventory (BAI) was administered to the participating patients 1 day before surgery to evaluate the level of anxiety. According to BAI 1–7 point minimal anxiety, 8–15 point mild anxiety, 16–25 point moderate anxiety and 26 and above severe anxiety. We took the cut-off value of 8 was taken as the mild anxiety. Based on the scores from the BAI, 40 patients with an anxiety score of < 8 were assigned to the non-anxious group (Group NA) and 40 patients with an anxiety score of ≥8 were assigned to the anxious group (Group A). The MPV in the complete blood count test performed 1 day prior to surgery was recorded. All patients entering the operation room routinely underwent electrocardiography, non-invasive blood pressure measurement, peripheral oxygen saturation (spO_2_) measurement, and entropy monitoring (GE entropy sensor, GE Healthcare, Finland).

Vascular access was attained on the dorsum of the left hand using an 18-gauge intravenous catheter and all patients premedicated with 0.02 mg/kg midazolam. A physiological saline was initiated at a rate of 15 mlkg^− 1^ h^− 1^. Anesthesia was induced through the administration of propofol 1% at a rate of 3 mg / kg /3 min using an infuser. The pain from the propofol infusion was rated as described elsewhere (0, none; 1, mild; 2, moderate; 3, severe) by an anesthesiologist who was blind to the study groups and treatments [9]. The mean time to achieve an entropy value below 60 (T1) was recorded and a remifentanil infusion was initiated for 2 min at a rate of 1 μg/kg^− 1^/min^− 1^. Rocuronium 0.6 mg/kg was administered for neuromuscular blockage and the Evans scores of the patients after intubation were recorded.

For the maintenance of anesthesia, intravenous propofol was continued at a rate of 3 to 7 mg/kg-^1^/h^− 1^ and the remifentanil infusion was continued at a rate of 0.1 μg/kg^− 1^/min^− 1^ to maintain the target entropy value of between 40 and 60. The remifentanil infusion rate was increased to 0.3 μg/kg^− 1^/min^− 1^, if the entropy value was above 60 with a propofol infusion rate of 7 mg/kg^− 1^/h^− 1^. Mechanical ventilation was continued with 3 L fresh gas flow containing 40% O_2_ and 60% air. All patients were administered intravenous dexketoprofen 50 mg and tramadol 1 mg/kg^− 1^ for preemptive analgesia. The total intraoperative propofol consumption within the first 30 min was recorded. The remifentanil infusion was ceased 10 min before completion of surgery, and the propofol infusion was ceased after suture placement. The time from the end of anesthesia to eye opening was recorded (T2). Along with these measurements, the mean arterial pressure (MAP), heart rate (HR), end-tidal carbon dioxide (etCO_2_), and spO_2_ were recorded before induction, before intubation, and at 5, 10, 30, 45, and 60 min after intubation.

### Statistical analysis

Previous studies in the literature were taken into consideration while estimating the sample size [10, 11]. It was hypothesized that a 20% difference in the preoperative MPV of patients with low and high anxiety scores would be of clinical significance. The minimum number of patients in each group was calculated as 34 considering an alpha (two-tailed) of 0.05 and a power of 0.80.

Statistical analysis was performed using the SPSS version 15.0 software (SPSS Inc., Chicago, IL, USA). The relationship between MPV and preoperative anxiety scores was evaluated using the Pearson’s correlation coefficient. A receiver operating characteristic (ROC) curve analysis was performed to evaluate the relationship between the BAI scores and MPV and propofol consumption at 30 min. A *p* value of less than 0.05 was considered statistically significant.

## Results

**Fig. 1:**
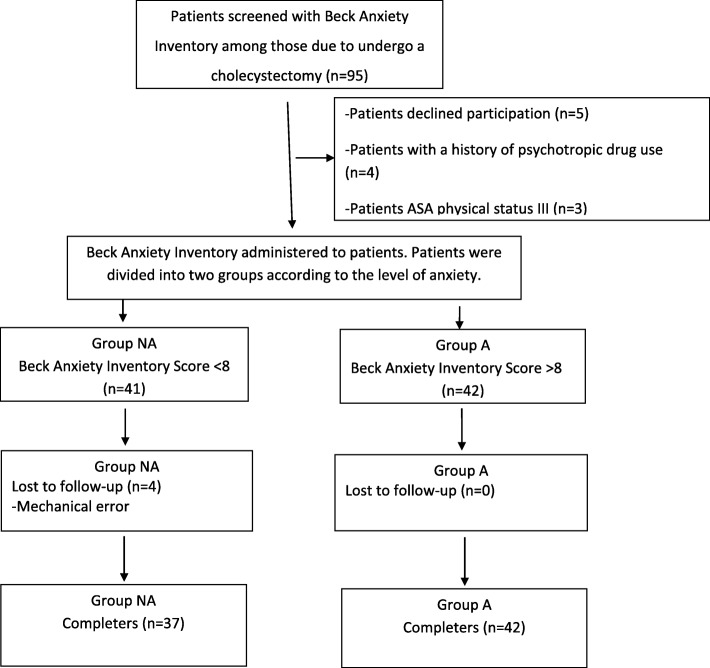
Study flow chart

A total of 95 patients were evaluated using the BAI, after which, five patients who did not give their consent for participation in the study, four with a history of psychotropic drug use, and three with ASA physical status III were excluded. Finally, 83 patients were divided into two groups based on their anxiety scores (Fig. 1).

The demographic data was presented Table 1. The age, sex, and MPV were significant difference between groups. The mean MPV in the preoperative complete blood count was 10.23 ± 1.42 in Group A and 8.98 ± 0.91 in Group NA (*p* = 0.02) (Table 1).

**Table 1:** Descriptive variables of group anxious and group non-anxious

	Group Anxious *n* = 42	Group Non-Anxious *n* = 37	*p*
Age	44.08 ± 15.88	36.09 ± 11.28	0.01
Sex (F/M)	22/15	14 / 28	0.02
BMI	23.90 ± 4.30	26.92 ± 4.40	0.28
MPV	10.23 ± 1.42	8.98 ± 0.91	0.02
Platelet count	278.85 ± 31.34	256.64 ± 48.65	0.10

Results are expressed as mean ± standard deviation or number of patients

*F* Female, *M* Male

In terms of intraoperative data, time to achieve an entropy value of below 60 after induction and the time to eye opening after surgery were similar in Group A and Group NA (*p* = 0.12 and *p* = 0.25, respectively). The mean total propofol consumption at 30 min after induction was 418.35 ± 121.98 mg in Group A and 344.04 ± 72.03 mg in Group NA. The difference between the groups was statistically significant (*p* = 0.001). The median value and the percentage of propofol injection pain in both groups are shown in Table 2.

**Table 2:** Comparison of intraoperative variables of group anxious and group non-anxious

	Group Anxious *n* = 42	Group Non-Anxious *n* = 37	*p*
Profofol injection pain^a^	1.00	1.50	0.06
Profofol injection pain^b^ (n,%)	None (13,%35.1)	None (7,%16.7)	
	Mild (13,%35.1)	Mild (14,%33.3)	
	Moderate (6,%16.2)	Moderate (13,%31.0)	
	Severe (5,13.5)	Severe (8,%19.0)	
Time to achieve an entropy value below 60 (sec)	128.51 ± 44.74	133.11 ± 34.00	0.12
Evans score	1.67 ± 1.56	1.66 ± 1.76	0.98
Propofol consumption at 30 min (mg)	418.35 ± 121.98	344.04 ± 72.03	0.001
Total propofol consumption (mg)	808.32 ± 256.39	650.45 ± 143.34	0.001
Total remifentanil consumption (mcg)	423.56 ± 91.30	426.07 ± 74.60	0.89
Time to eye opening (sec)	261.56 ± 120.88	224.90 ± 156.02	0.25
Operation time (min)	57.70 ± 6.30	55.66 ± 9.70	0.28

The other results are expressed as mean ± standard deviation

^a^Propofol injection pain is expresses as median

^b^The percentage of Propofol injection pain

**Fig. 2:**
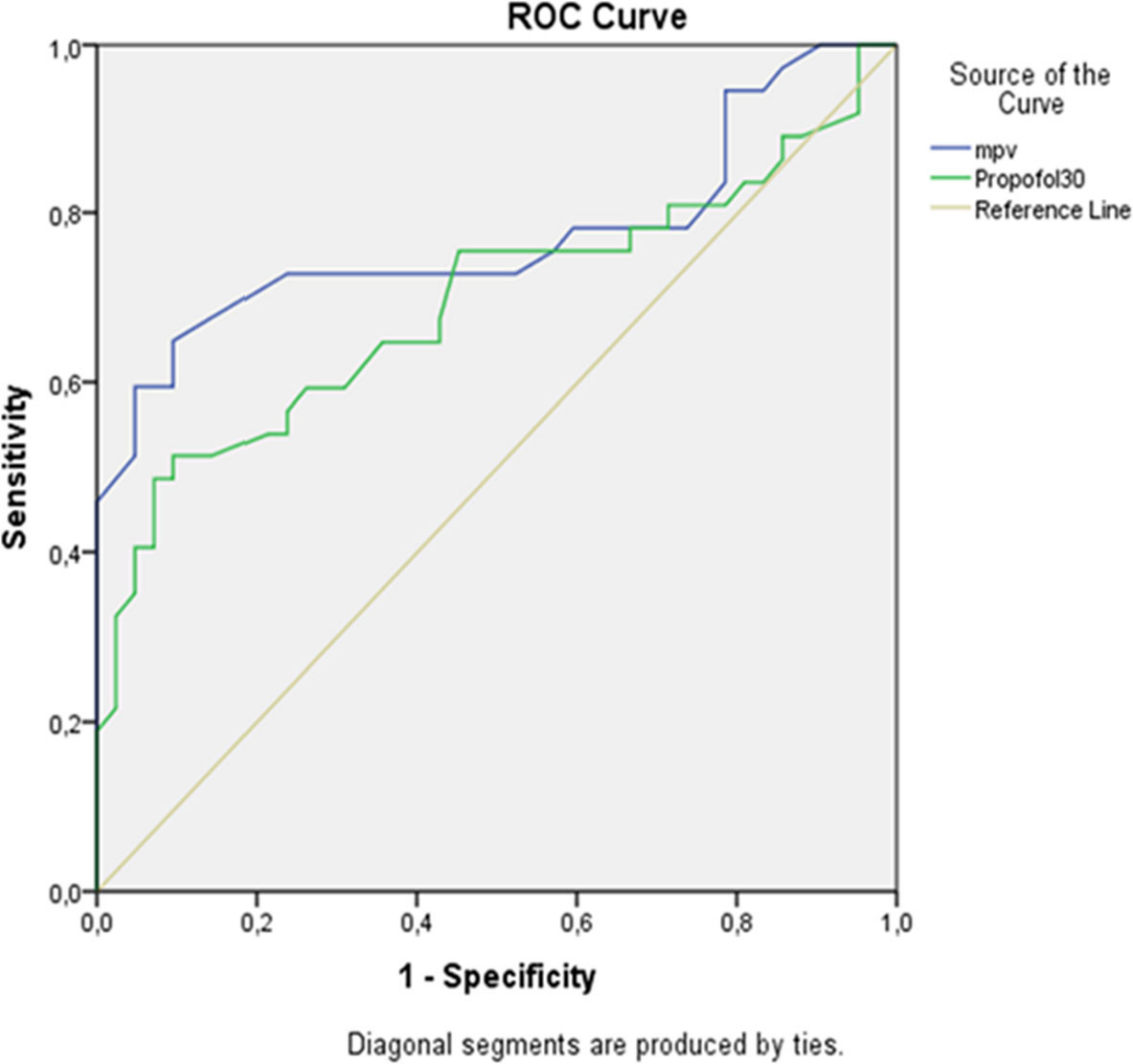
ROC curve analysis and area under the curve (AUC) showing the relationship between the Beck score and MPV and propofol consumption at 30 min

According to the Pearson’s correlation analysis, we found a correlation between the MPV and anxiety scores in the anxious group (*r* = 0.336, *p* = 0.04), whereas no correlation was found in the non-anxious group (*r* = 0.126; *p* = 0.42). The area under curve (AUC) for MPV was recorded as 77.3% with 5.8% standard deviation. AUC for Propofol was 69.5% with 6.2% standard deviation. The ROC curve analysis showed that the best sensitivity value was 73% and the specificity value was 76%. Accordingly, the cut-off value for MPV was calculated as 9.65 (Fig. 2).

## Discussion

In the present study, we found significantly higher preoperative MPV values among patients with high preoperative anxiety scores. Similarly, propofol consumption at 30 min was higher in patients with higher anxiety scores. However, we found no relationship between the time to achieve entropy value below 60 and anxiety scores.

Psychological factors have an effect on anesthetic requirements and postoperative pain in patients undergoing a selected surgical procedure. Kil et al. [10] investigated the effects of anxiety on the amount of propofol to achieve different levels of sedation. In addition, a high anxiety level increased the propofol requirement for both superficial sedation (BIS 85) and moderate sedation (BIS 75). Propofol requirements for deep sedation (BIS 65) were higher with chronic anxiety, whereas no significant difference was noted in relation to state anxiety. In another study, Maranets et al. [7] reported that the baseline anxiety level increased the intraoperative analgesic requirement and, similarly, Hong et al. [8] showed a remarkable correlation between the amount of propofol required for sedation and the level of anxiety. In the present study, the time required for anesthesia induction using a constant rate of infusion of propofol was similar for patients with and without anxiety, whereas propofol consumption at 30 min was significantly higher in the anxious group.

There are several studies in the literature evaluating the effects of age and sex on anxiety. Kil et al. [10] showed that advancing age was not related to anxiety; however, they reported a relationship with increasing pain sensitivity. Another study [12] reported higher anxiety levels in female patients, and similar to previous studies, there was a female predominance in the group of patients with higher anxiety scores. Different from the literature, however, the mean age was significantly higher in patients with a high anxiety score. There are also some studies reporting that age and gender differences can effect on platelet functions [13–15].

Previous studies also demonstrated a relationship between increased platelet activity and anxiety and depression. In a study of 15 patients, Canan and Ataoğlu [12] reported elevated MPV values in patients with major depression, and also a marked decrease in the MPV values of eight patients undergoing escitalopram therapy. Another study comparing 289 patients with major depression and healthy control subjects reported higher MPV values in the patient group [16]. Multiple changes may occur in platelet parameters in patients with anxiety disorders [17]. Increased MPV values may be indicative of increased platelet activation or the presence of enlarged and extremely flexible platelets [18]. The possible mechanisms underlying platelet abnormalities in major depression are considered to be caused by changes in the platelet functions associated with alterations in intraplatelet monoamine and catecholamine concentrations, increases in plasma 5-HT and epinephrine concentrations, and increases in intraplatelet calcium mobilization [19]. The underlying mechanism in anxiety disorders has been associated with serotonin mechanism. Vizioli et al. [20] showed that increased sympathetic activity could result in an increase in MPV values. Serotonin 5-HT2A receptors and serotonin transporter receptors in the platelets and brain are encoded by the same gene [21], and as a result, serotonin-related platelet activation was reported in patients with anxiety disorder [21]. As opposed to other studies, Gul et al. [22] found lower MPV values in patients with anxiety disorder than in the control group. Although the authors were unable to explain the actual mechanism, they suggested that abnormal 5-HT metabolism in patients with panic disorder might have led to their finding.

The MPV values were higher in patients with high anxiety scores in the present study. The calculation of the area under curve in the ROC analysis showed that preoperative MPV values performed better in detecting the presence of anxiety than the level of propofol consumption. A cut-off MPV value of 9.65 yielded the best sensitivity (73%) and specificity (46%) values. In our study the cut off value of BAI is accepted as mild anxiety. If another study took this cut off value moderate anxiety there may be a higher correlation between MPV and anxiety.

The anxiety has an effect on the adrenergic system [14]. An increase in the adrenergic system results in an increase in platelet volume during the production processes. In other words, patients with high levels of anxiety generally have platelets with large volumes. We think that the mechanism of adrenergic system and platelet production process can explain the correlation between MPV and anxiety.

The main limitation of the present study is that age and sex, which may affect platelet function and anxiety, were not taken into consideration when recruiting participants, and as a result, a difference was observed between the groups in terms of age and gender. Studies of more homogeneous groups involving single age and sex groups may eliminate the possible effects of age and sex on MPV values.

## Conclusions

MPV is a cost-effective, fast, and easy parameter for the detection of preoperative anxiety and, based on our study results, we suggest that it is helpful in the clinical practice in predicting the amount of anesthetic agents required for the first 30 mins of anesthesia.

## Data Availability

The datasets used and/or analyzed during the current study are available from the corresponding author on reasonable request.

## Abbreviations

ASA: American Society of Anesthesiology
AUC: Area Under Curve
BAI: Beck Anxiety Inventory
BMI: Body Mass Index
etCO_2_: End-Tidal Carbon Dioxide
Group A: Anxious Group
Group NA: Non-Anxious Group
HR: Heart Rate
MAP: Mean Arterial Pressure
MPV: Mean Platelet Volume
ROC: Receiver Operating Characteristic
spO_2_: Peripheral Oxygen Saturation
T1: The mean time to achieve an entropy value below 60
T2: The time from the end of anesthesia to eye opening was recorded
